# A randomised active-controlled trial to examine the effects of an online mindfulness intervention on executive control, critical thinking and key thinking dispositions in a university student sample

**DOI:** 10.1186/s40359-018-0226-3

**Published:** 2018-04-05

**Authors:** Chris Noone, Michael J. Hogan

**Affiliations:** School of Psychology, National University of Ireland Galway, Newcastle Road, Galway, Ireland

## Abstract

**Background:**

Arguments for including mindfulness instruction in higher education have included claims about the benefits of mindfulness practice for critical thinking. While there is theoretical support for this claim, empirical support is limited. The aim of this study was to test this claim by investigating the effects of an online mindfulness intervention on executive function, critical thinking skills and associated thinking dispositions.

**Method:**

Participants recruited from a university were randomly allocated, following screening, to either a mindfulness meditation group or a sham meditation group. Both the researchers and the participants were blind to group allocation. The intervention content for both groups was delivered through the Headspace online application, an application which provides guided meditations to users. Both groups were requested to complete 30 guided mindfulness meditation sessions across a 6 week period. Primary outcome measures assessed mindfulness, executive functioning, critical thinking, actively open-minded thinking and need for cognition. Secondary outcome measures assessed wellbeing, positive and negative affect, and real-world outcomes.

**Results:**

In a series of full-information maximum likelihood analyses, significant increases in mindfulness dispositions and critical thinking scores were observed in both the mindfulness meditation and sham meditation groups. However, no significant effects of group allocation were observed for either primary or secondary measures. Furthermore, mediation analyses testing the indirect effect of group allocation through executive functioning performance did not reveal a significant result and moderation analyses showed that the effect of the intervention did not depend on baseline levels of the key thinking dispositions, actively open-minded thinking and need for cognition.

**Conclusion:**

No evidence was found to suggest that engaging in guided mindfulness practice for 6 weeks using the online intervention method applied in this study improves critical thinking performance. While further research is warranted, claims regarding the benefits of mindfulness practice for critical thinking should be tempered in the meantime.

**Trial registration:**

The study was initially registered in the AEA Social Science Registry before the recruitment was initiated (RCT ID: AEARCTR-0000756; 14/11/2015) and retrospectively registered in the ISRCTN registry (RCT ID: ISRCTN16588423) in line with requirements for publishing the study protocol.

**Electronic supplementary material:**

The online version of this article (10.1186/s40359-018-0226-3) contains supplementary material, which is available to authorized users.

## Background

Mindfulness has been operationalised as a mental state involving two components: the self-regulation of attention so that thoughts, feelings and sensations are observed and attended to in the present-moment and an orientation to experience characterised by acceptance, non-judgment and non-reactivity [1]. It has been claimed that mindfulness should facilitate critical thinking in higher-education, based on early Buddhist conceptualisations of mindfulness as clarity of thought [2]. There is theoretical support and some empirical evidence for this claim, such as in a recent cross-sectional study where evidence for inhibition mediating a positive relationship between dispositional mindfulness and critical thinking was demonstrated [3] but it remains an open question as to whether mindfulness practice enhances critical thinking or not (or even hinders it; [4]). It is important to test the veracity of claims regarding mindfulness and critical thinking in the most rigorous way available. To achieve this, a randomised controlled trial was conducted to compare the effects of mindfulness training on executive function and critical thinking to those of a closely matched active control condition.

Common to most conceptualisations of critical thinking in psychology is the need to evaluate arguments and evidence without influence from one’s own prior belief and experience [5]. Critical thinking involves the effective use of the cognitive skills of analysis, evaluation, and inference, in a purposeful, reasoned and goal-directed manner [6]. Halpern’s [6] description of the higher-order thinking skills involved in critical thinking includes verbal reasoning, argument analysis, hypothesis testing, estimating likelihood and dealing with uncertainty, problem solving, and decision-making. In her definition of critical thinking, its function is the selection of thinking strategies which increase the probability of a desirable outcome [7]. Thus the defining features of critical thinking are not the characteristics of the thinking skills employed but the process of selecting and executing the appropriate thinking skill and the monitor and control of this thinking process [6, 8, 9]. The appropriate execution of these critical thinking skills depends on the presence of specific dispositions towards thinking with two in particular being the focus of much research – need for cognition and actively open-minded thinking [5, 10–12]. The application of critical thinking skills also depends on the thinker’s awareness that a particular thinking skill is required, that the ongoing execution of the skill is adequate, and the ability to monitor and exert control to change ongoing thinking processes [8, 9, 13, 14]. Performance on measures of critical thinking and tasks assessing heuristic and biased thinking has been positively associated with better real-world outcomes [15–24].

The rationale for this study relies on previous studies suggesting positive effects of mindfulness on aspects of executive functioning [25, 26] and higher-order cognition [3, 27–30] and a specific type of default-interventionist dual-process theory (i.e. the three-stage dual-process model of analytic engagement) which can act as a framework to integrate research on the effects of mindfulness on executive function and the self-regulation of thinking and decision-making [31]. Dual-process theories of cognition posit that cognitive processes can be organised into two categories: type-1 processes and type-2 processes. Type-1 processes are those which are generally fast, automatic and do not require working memory. Current evidence suggest that they occur by default in response to stimuli (due to either innate or learned tendencies) and resulting models of cognition are referred to as default-interventionist accounts. Type-2 processes are generally slow and controlled. Their defining feature is the representation and comparison of hypothetical models of the world which requires working memory resources. When presented with a given stimulus or situation, different cognitive simulations of possible actions are compared to test their appropriateness, before acting according to the preferred response [32, 33]. In most situations (i.e. those where the processes have not been automatized through experience), all higher-order thinking skills such as problem-solving, decision-making and critical thinking, involve type-2 processes. Since Type-1 processes occur by default, they must be overridden to allow the engagement of Type-2 processes. The control processes which achieve this are referred to as executive functions and the process of inhibition is particularly important [5, 34–36]. Inhibition is the process by which prepotent responses and habitual behaviours are overridden, thus allowing for other responses [35]. In line with this theory, individual differences in executive functioning tend to account for a significant share of the variance in higher-order thinking skill [37, 38]. Furthermore, there is much experimental evidence to support this model [39–41]. This model provides a useful theoretical framework for the current study for two reasons. First, mindfulness practice has been shown to be associated with improvements in executive functioning [25, 42]. A cognitive model of mindfulness proposed by Teper and colleagues [43] outlines the mechanisms which may connect mindfulness practice to the intervention of executive functioning in the dual-process model described above. This model suggests that the present-moment attention or observation cultivated during mindfulness practice allows for the detection of affective cues which are typically not noticed. This allows negative affective cues which serve as triggers for self-regulation to be noticed. These negative affective cues carry information indicating that an individual’s current state is inconsistent with their goal state. The model also implies that a mindful orientation of acceptance or non-reactivity involves inhibiting automatic tendencies to elaborate upon affective cues. Crucially, conflict between default type-1 responses has been shown to produce negative affective cues which are thought to trigger type-2 processes [41, 44–46]. Taken together, these sets of findings imply that present-moment attention may facilitate the detection of negative affective cues produced by the conflict of type-1 responses. They also imply that non-reactivity may facilitate the inhibition of type-1 responses required for type-2 processing to intervene. Second, studies showing improvements in certain aspects of higher-order thinking generally explain their results by claiming that mindfulness practice has trained participants to inhibit automatic, or type-1, processing [27, 29, 47, 48]. Notably, a recent cross-sectional study focusing on individual differences in mindfulness, executive functioning and critical thinking supported this model by demonstrating evidence for skill in inhibition mediating a positive relationship between mindfulness and critical thinking [3].

Advances in technology are allowing the design of mindfulness interventions with more experimental control than previously possible [49]. The development of smartphone and web applications focused on the delivery of guided meditations in particular has made it easier to implement rigorous designs by facilitating the inclusion of objective measures of time spent meditating and by reducing the resources needed for running an intervention as well as the demands placed on the participants. Previous studies involving smartphone delivery of mindfulness interventions focused on workplace stress [50], wellbeing [49], depression [51] and compassion [52]. A recent meta-analysis showed that online mindfulness interventions tend to yield comparable results to traditional interventions focused on similar outcome variables such as stress, depression, anxiety and wellbeing, with effect sizes ranging from *g* = 0.22 to *g* = 0.51 [53]. These studies can also be considered more rigorous due to the standardisation of instruction across participants in the experimental group and the use of objective measures of adherence to the intervention (provided through the app) rather than self-report [54]. However, these studies would have been strengthened further by the inclusion of more closely matched active-control materials as has been done in offline studies [55, 56]. The current study takes this approach by using the same interface, the Headspace application, to deliver sham intervention content which participants could reasonably believe is mindfulness training [57, 58].

### The current study

The central research question for this study was “does regular mindfulness meditation practice facilitate critical thinking through the enhancement of executive function?” A secondary research question focused on whether regular mindfulness meditation practice enhance critical thinking dispositions. Thinking dispositions are key determinants of critical thinking which may interact with mindfulness practice [59]. In addressing these questions, this study employed a randomised controlled trial which compared a 6-week online mindfulness meditation intervention to a 6-week online sham meditation intervention, which acted as an active-control condition. For a list of the study hypotheses, see Table 1.

**Table 1:** Study Hypotheses

Outcomes	Variable	Measure		Hypothesis
Primary	Mindfulness	Five Facet Mindfulness Questionnaire	1	Mindfulness will increase more for the mindfulness meditation (MM) group than for the sham meditation (SM) group from baseline to follow-up
	Critical Thinking	Halpern Critical Thinking Assessment^1^, Heuristic and Biases items^2^	2	Critical thinking will increase more for the MM group than for the SM group from baseline to follow-up *(a* ^*1,2*^*)* and this effect will be moderated by baseline endorsement of thinking dispositions *(b*^*1,2*^*)*
	Thinking Dispositions	Actively Open-minded Thinking^1^, Need for Cognition^2^	3	Endorsement of critical thinking dispositions will increase more for the MM group than for the SM group from baseline to follow-up *(a* ^*1,2*^*)*
	Executive Control	Sternberg Working Memory Task	4	Executive control will increase more for the MM group than for the SM group from baseline to follow-up *(a)* and this increase will mediate the relationship between levels of mindfulness and critical thinking performance following the intervention *(b)*
Secondary	Wellbeing	Warwick-Edinburgh Mental Wellbeing Scale	5	Wellbeing will increase more for the MM group than for the SM group from baseline to follow-up
	Positive Affect and Negative Affect	Positive Affect and Negative Affect Schedule subscale	6	Positive affect will increase more and negative affect will decrease for the MM group than for the SM group from baseline to follow-up *(a)*
	Real-world Outcomes	Real-world Outcomes Inventory	7	Negative real-world outcomes will decrease more for the MM group than for SM group from baseline to follow-up
Manipulation Checks	Meditation Quality	Practice Quality-Meditation	8	Meditation quality will be positively associated with increases in mindfulness *(a)*, executive control *(b)* and critical thinking *(c* ^*1,2*^*)* and meditation quantity *(d)*, task enjoyment *(e)* and task difficulty *(f)* and it will be higher in the MM group and across time.
	Meditation Quantity	Total Number of Completed Meditation Sessions	9	Meditation quantity will be positively associated with increases in mindfulness *(a)*, executive control *(b)* and critical thinking *(c* ^*1,2*^*)* and meditation quality *(d)*, task enjoyment *(e)* and task difficulty *(f)* and will not differ across groups.
	Task Enjoyment	Technology Acceptance Model Questionnaire Perceived Enjoyment subscale	10	Task enjoyment will be positively associated with increases in mindfulness *(a)*, executive control *(b)* and critical thinking *(c 1,2*) and meditation quality *(d)*, meditation quantity *(e)* and task difficulty *(f)* and will not differ across time or groups.
	Task Difficulty	Technology Acceptance Model Questionnaire Perceived Ease subscale	11	Task difficulty will be positively associated with increases in mindfulness *(a)*, executive control *(b)* and critical thinking *(c 1,2*) and meditation quality *(d)*, meditation quantity *(e)* and task difficulty *(f)* will not differ across time or groups.
	Intervention Acceptability	Satisfaction items from Kirkpatrick et al. (2013) [84]	12	Intervention acceptability will be positively associated with increases in mindfulness *(a)*, executive control *(b)* and critical thinking *(c* ^*1,2*^*)* and meditation quantity *(d)*, task enjoyment *(e)* and task difficulty *(f)* and it will be higher in the MM group but will not differ across time.
	Attrition	No. of participants lost from baseline to follow-up	13	Attrition will be negatively associated with meditation quality *(a)*, meditation quantity *(b),* task enjoyment *(c)* and task difficulty

Many universities and other institutions are introducing mindfulness programmes with the promise of improving thinking skills [60]. While there are theoretical and historical reasons supporting this view, it has not been adequately investigated and so this claim is premature. The contribution of this study lies in its rigorous approach to investigating this claim for the first time in the context of a randomised controlled trial (RCT).

## Methods

### Design

**Fig. 1:**
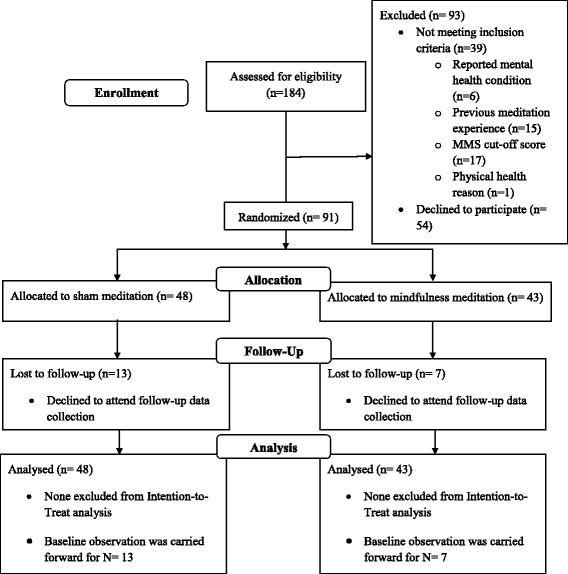
CONSORT [105] flowchart of progress through phases of the current study. Sample size (Incl. flow chart)

This pre-registered study involved a two-arm randomised-controlled superiority trial with one intervention condition, guided mindfulness meditation, and one active-control condition, sham meditation. The design employed was a 2 (condition) × 2 (time) parallel-group design which is explanatory in nature. Baseline measurement took place immediately before randomisation and follow-up measurement took place 6 weeks after the beginning of the intervention. The content of both the intervention condition and the active-control condition was delivered via a smartphone/online application between baseline and follow-up. Manipulation checks were carried out to assess intervention acceptability, technology acceptance and meditation quality 2 weeks after baseline and 4 weeks after baseline. See Fig. 1 for a flowchart including the procedure. The protocol for this study was published in advance of its completion [61] and both the protocol and study are reported according to CONSORT guidelines [62]. No changes were made to the conduct of the trial following this but the analytic approach has been amended following advice from a reviewer.^1^

An a priori sample size calculation carried out using G*Power [63] for the original analytic approach revealed that with 2 groups, 4 measurements, an assumed correlation among repeated measures of 0.3 (typically low in such research; Rossi [64]) as well as a medium effect size (again typical in research on the cognitive effects of mindfulness; Chiesa et al. [42]) and a power of 0.8, the recommended sample size for mixed (repeated-measures and between factors) ANOVA was 56. We expected an attrition rate of 20% from baseline to follow-up based on reported attrition rates of between 20 and 40% [49, 52] for research using the Headspace application and the incentives available in the form of course credit, lunches provided at data collection and free subscription to Headspace for six months following the intervention. With this in mind, we sought to recruit at least 80 participants. Following screening, our baseline sample included 91 participants and attrition led to a follow-up sample of 71 participants. Fig. 1 depicts the flow of participants through the study.

### Sample characteristics

Table 2 presents the characteristics of our sample. Our inclusion criteria specified that university students at NUI Galway who are over 18 years of age, below 65 years of age and have either English as first language or university level English (i.e. equivalent to 80 on TOEFL or 6.5 on IELTS) were eligible for this study.

**Table 2:** Sample Characteristics

	Sham Meditation		Mindfulness Meditation		Overall	
	Baseline	Follow-up	Baseline	Follow-up	Baseline	Follow-up
*N*	48	35	43	36	91	71
Females/Males^a^	35/13	27/8	34/7	29/5	69/20	56/13
Age – *M (SD)*	21.06 (4.67)	20.74 (3.43)	20.77 (4.11)	20.39 (3.64)	20.92 (4.39)	20.56 (3.52)
Years in Higher Education – *M (SD)*	1.93 (2.01)	1.87 (2.02)	1.37 (0.93)	1.33 (0.89)	1.66 (1.61)	1.60 (1.57)

^a^Two participants chose not to report their gender

The exclusion criteria included previous experience in meditation, alcohol or drug dependence, current use of sedating medication, history of medical conditions associated with a head injury, spinal injury, epilepsy, or stroke, lack of normal or corrected-to-normal vision and hearing and current experience of high levels of depression, anxiety or psychotic symptoms. This last criterion was assessed with the Modified Mini Screen (OASAS [65]). Phone calls were made to debrief people who were excluded and they were offered access to the intervention materials. If any potential participants had exceeded the cut-off score on the Modified Mini Screen, an experienced clinician was available for support.

### Randomisation

Participants accessed the intervention content relevant to their group allocation using a unique code provided by Headspace staff. These codes were initially provided to the researchers in lists labelled “Condition A” and “Condition B”. Participants were randomly allocated to the Condition A or Condition B using block randomisation with a 1:1 ratio and a fixed block of 6 and then given their code by the researcher [66]. Following the completion of data analysis, the Headspace staff informed the researchers which condition the mindfulness meditation group was (i.e. A or B) and which condition the sham meditation group was. Through this procedure, double-blinding was achieved. If it had been necessary for any reason, blinding could have been undone on an individual basis through dialogue between the researchers and the Headspace staff.

### Intervention

All of the intervention content was delivered through the Headspace application. This application can run on any iOS or Android smartphone or tablet and through internet browsers. Once participants had set up an account and entered their unique randomisation code, they could follow a guided meditation session whenever and wherever suited them. The intervention was 6 weeks in duration and participants were requested to complete 30 meditation sessions during this time. Each session lasted 10 min. The sole difference in the experience for participants in the two study groups was the nature of the guided sessions, as described next.

### Experimental condition

The content of the initial sessions in this condition focused on introducing the concept of mindfulness, practical tips for practicing mindfulness meditation and a guided body-scan meditation. Later sessions introduced a breath counting exercise during the guided meditations and developed a greater emphasis on non-judgmental awareness. For a session-by-session description, see Additional file 1. This content was developed and delivered by Andy Puddicombe who is an internationally recognised expert in mindfulness practice and teaching.

### Active-control condition

This condition presented the participants with guided breathing exercises. Each session began by inviting the participants to sit with their eyes closed. These exercises were referred to as meditation but participants were not given guidance on how to control their awareness of their body or breath. This approach was designed to control for the effects of expectations surrounding mindfulness and physiological relaxation to ensure that the effect size could be attributed to mindfulness practice specifically. This content was also delivered by Andy Puddicombe and was developed based on previous work by Zeidan and colleagues [55, 57, 58].

### Data collection

Baseline data was collected during the week preceding the beginning of the intervention. Follow-up data was collected during the week following the end of the intervention. The data was collected in the PC suite of the NUI Galway School of Psychology. The Sternberg Working Memory was presented using Inquisit [67] and the remaining measures were presented using SurveyGizmo. No changes were made to the outcome measures used following initial design and registration.

### Primary outcome measures

Reliability analysis for the questionnaires used in this study was conducted using the Scale Diagnosis function from the UserFriendlyScience package in R which allows the examination of Cronbach’s alpha (α), omega (ω) and the greatest lower bound (GLB) [68]. Reliability for each 2-item factor is computed using the Spearman-Brown coefficient [69].

#### Halpern Critical Thinking Assessment (HCTA; [70])

The HCTA involves 25 real-world involving medical research, social policy analysis and other types of problems encountered in everyday life. Each situation is accompanied by both open and closed questions. A standardised guide answers is used to score forced-choice questions. This guide includes specific scoring prompts for open-ended questions (for more detail see [71]). The total possible score across all situations is 194 [70]. The internal reliability of the HTCA is usually adequate [15, 71] and was found to be so at both time points in this study (Baseline: Cronbach’s α = 0.72 [0.64, 0.80], ω = 0.73 [0.65, 0.81], GLB = 0.79; Follow-up: Cronbach’s α = 0.81 [0.74, 0.87], ω = 0.81 [0.75, 0.88], GLB = 0.79).

#### Heuristics and Biases items [5]

These 16 items were taken from the literature on judgment and decision-making. It has been suggested that they assess aspects of critical thinking not captured by traditional measures (for more detail see [5, 61]). Each of these items was scored as either correct or incorrect so total score of 16 was possible. Though these items do not represent a unifactorial construct, we followed West and colleagues [5] in aggregating the scores on these items and as a result found poor reliability (Baseline: Cronbach’s α = 0.60 [0.49, 0.72], ω = 0.61 [0.50, 0.72], GLB = 0.67; Follow-up: Cronbach’s α = 0.34 [0.24, 0.54], ω = 0.46 [0.36, 0.56], GLB = 0.66) which suggests that multiple processes underlie the rational thinking required by these items. Common to these items, however, is the need to inhibit an automatic heuristic response and this is the process of interest in this study.

#### Five Facet Mindfulness Questionnaire (FFMQ; [72])

The FFMQ includes 39 items across 5 sub-scales tapping separate facets of dispositional mindfulness: describing (Baseline: Cronbach’s α = 0.89 [0.85, 0.92], ω = 0.89 [0.86, 0.92], GLB = 0.93; Follow-up: Cronbach’s α = 0.90 [0.86, 0.93], ω = 0.90 [0.86, 0.93], GLB = 0.94), observing (Baseline: Cronbach’s α = 0.75 [0.67, 0.83], ω = 0.76 [0.68, 0.83], GLB = 0.87; Follow-up: Cronbach’s α = 0.82 [0.76, 0.88], ω = 0.83 [0.77, 0.89], GLB = 0.90), non-reactivity (Baseline: Cronbach’s α = 0.82 [0.76, 0.87], ω = 0.82 [0.77, 0.88], GLB = 0.91; Follow-up: Cronbach’s α = 0.77 [0.68, 0.85], ω = 0.77 [0.69, 0.85], GLB = 0.84), non-judgment (Baseline: Cronbach’s α = 0.88 [0.84, 0.92], ω = 0.88 [0.85, 0.92], GLB = 0.90; Follow-up: Cronbach’s α = 0.92 [0.89, 0.95], ω = 0.92 [0.89, 0.95], GLB = 0.96) and acting with awareness (Baseline: Cronbach’s α = 0.86 [0.82, 0.91], ω = 0.86 [0.82, 0.91], GLB = 0.93; Follow-up: Cronbach’s α = 0.87 [0.82, 0.91], ω = 0.87 [0.82, 0.91], GLB = 0.95). Responses are captured on a 5-point Likert scale (e.g. 1 = never or very rarely true; 5 = very often or always true). It has been shown to have adequate internal consistency and construct validity [72].

#### Sternberg working memory task [73]

This task is a measure of executive control of working memory. Participants were required to memorise a series of letters. They then indicated, as quickly and accurately as possible, whether a probe was in this series. There were 54 trials and the number of accurate responses was employed as the dependent variable. This task was used as it had been successfully applied using the Inquisit online experiment software in a previous study on mindfulness and executive control [26] and since it assesses working memory resources which, as described above, are necessary for the engagement of type-2 processes such as critical thinking.

### Secondary outcome measures

#### Positive and Negative Affect Schedule (PANAS; [74])

This scale was used to assess general levels of positive and negative affect by asking participants to indicate to what extent they felt each of 20 positive and 10 negative emotions over the past week using a 5-point Likert scale (e.g. 1 = very slightly or not at all; 5 = extremely). This scale tends to demonstrate good reliability [75] and this was replicated in the current study for the positive (Baseline: Cronbach’s α = 0.87 [0.83, 0.91], ω = 0.87 [0.83, 0.91], GLB = 0.94; Follow-up: Cronbach’s α = 0.90 [0.86, 0.93], ω = 0.90 [0.86, 0.93], GLB = 0.95) and negative affect subscales (Baseline: Cronbach’s α = 0.86 [0.82, 0.91], ω = 0.87 [0.83, 0.91], GLB = 0.92; Follow-up: Cronbach’s α = 0.86, ω = 0.86 [0.82, 0.91], GLB = 0.92).

#### Warwick-Edinburgh mental wellbeing scale [76]

This 14 item scale assesses subjective well-being and psychological functioning. The scale is scored by summing responses to each item answered on a 5 point Likert scale. The total possible score is therefore 70 and a high score reflects a high level of positive mental health [77]. This scale showed excellent reliability in this study (Baseline: Cronbach’s α = 0.85 [0.78, 0.88], ω = 0.86 [0.78, 0.88], GLB = 0.94; Follow-up: Cronbach’s α = 0.89 [0.86, 0.93], ω = 0.90 [0.86, 0.93], GLB = 0.95). The wellbeing measures were included in order to allow comparison between this study and previous studies which had employed a similar online intervention method to manipulate mindfulness practice.

#### Real world outcomes inventory [16]

This is a behavioural checklist focused on negative life outcomes from many domains ranging in severity. It has been shown to be negatively associated with critical thinking (i.e. higher critical thinking performance related to fewer negative outcomes). It was slightly adapted to ensure cultural relevance by omitting items which do not fit the Irish student context (e.g. got blisters from sunburn). The checklist presented participants with 32 possible outcomes and they were asked to indicate whether they had experienced each outcome in the previous 2 weeks.

#### Adherence

Objective adherence data was collected through the Headspace accounts of the participants. The number of completed sessions was recorded.

### Potential moderators

#### Need for cognition scale [78]

This unidimensional scale assesses individuals’ tendency to engage in effortful cognitive activity [78]. The scale includes 18 items which are rated on a 5-point Likert scale (e.g. 1 = extremely uncharacteristic of me; 5 = extremely characteristic of me) and has a total possible score of 90. It has been extensively validated and has been found to have adequate reliability [79]. It had excellent reliability in this study (Baseline: Cronbach’s α = 0.89 [0.85, 0.92], ω = 0.89 [0.86, 0.92], GLB = 0.95; Follow-up: Cronbach’s α = 0.90 [0.87, 0.93], ω = 0.98 [0.87, 0.94], GLB = 0.92).

#### Actively open-minded thinking scale [80]

This scale assesses the extent to which individuals tend to approach information in an open and flexible manner. It includes 41 items which are rated on a 6-point Likert scale (e.g. 1 = strongly agree; 6 = strongly disagree). The total possible score is 246. It has been validated as unidimensional and is found to be reliable [81]. It demonstrated adequate reliability in this study (Baseline: Cronbach’s α = 0.87 [0.83, 0.91], ω = 0.87 [0.83, 0.91], GLB = 0.86; Follow-up: Cronbach’s α = 0.89 [0.85, 0.92], ω = 0.89, [0.85, 0.92], GLB = 0.89).

### Manipulation checks

Participants were asked to complete these manipulation checks online directly following a guided meditation session. A survey containing the following measures was sent to participants’ by email at 2 and 4 weeks following the start of the intervention.

#### Practice quality - mindfulness questionnaire [82]

This 6 item questionnaire consists of two factors assessing perseverance (i.e. persistent returning of focus to object of meditation) and receptivity (i.e. a willingness to embrace the experience) during meditation. Participants indicated the percentage of time during their meditation session that day during which their experience reflected each of the item statements. This scale has been shown to fit a 2-factor structure and practice quality predicts improvements in psychological symptoms [82]. Both the perseverance (Week 2: Cronbach’s α = 0.77 [0.62, 0.91], ω = 0.79 [0.67, 0.91], GLB = 0.86; Week 4: Cronbach’s α = 0.67 [0.55, 0.79], ω = 0.79 [0.58, 0.80], GLB = 0.73) and receptivity (Week 2: Cronbach’s α = 0.81 [0.69, 0.93], ω = 0.81 [0.70, 0.93], GLB = 0.82; Week 4: Cronbach’s α = 0.74 [0.64, 0.83], ω = 0.77 [0.69, 0.84], GLB = 0.85) subscales showed adequate reliability.

#### Technology Acceptance Model questionnaire (TAM; [83])

The TAM was employed to assess participants’ perceptions regarding their use of the Headspace app. The scale consists of factors assessing barriers to use (3 items; Week 2: Cronbach’s α = 0.88 [0.84, 0.92], ω = 0.90 [0.86,0 .94], GLB = 0.92; Week 4: Cronbach’s α = 0.87 [0.82, 0.92], ω = 0.88 [0.84, 0.92], GLB = 0.73), perceived ease of use (3 items; Week 2: Cronbach’s α = 0.89 [0.82, 0.96], ω = 0.90 [0.85, 0.96], GLB = 0.91; Week 4: Cronbach’s α = 0.74 [0.64, 0.83], ω = 0.77 [0.69, 0.85], GLB = 0.79), enjoyment (2 items; Week 2: Spearman-Brown = 0.92; Week 4: Spearman-Brown = 0.87) and intention to use (2 items; Week 2: Spearman-Brown = 0.96; Week 4: Spearman-Brown = 0.95). Items are measured on a 5-point Likert scale (e.g. 1 = strongly disagree; 5 = strongly agree).

#### Intervention acceptability [84]

Participants were asked about their overall satisfaction with the intervention and their satisfaction with the guided session content in particular. They were also asked binary questions about whether they would recommend the intervention to a friend and whether it was worth their time. These specific questions have been used to assess the acceptability of a range of low-intensity online interventions [84].

### Statistical analysis

Hypotheses regarding change in primary and secondary outcome measures from baseline to follow-up were tested in a series of regressions estimated using a full information maximum likelihood approach. These models examined the effect of group assignment on each measure at follow-up while controlling for baseline measurements. These analyses were conducted using AMOS [85]. Correlations between manipulation check measures were also examined as were their correlations with FFMQ change scores. These analyses were completed using SPSS 20 [86]. Simple mediation analyses were conducted using Structural Equation Modelling (SEM) to test whether executive function, meditation quality and adherence are mediators of any potential relationship between mindfulness and critical thinking. These analyses were also conducted using AMOS [85]. As noted above, these tests will be adequately powered – including SEM analyses (see Iacobucci et al. [87], for evidence of adequate power for simple mediation using SEM in samples as small as *n* = 30). See Table 1 for the specific tests employed for each hypothesis.

## Results

### Descriptive statistics and data inspection

Means and standard deviations for each dependent variable are displayed in Table 3. The data were inspected to ensure assumptions for the planned analyses were met. Q-Q plots, histograms and skewness and kurtosis values were examined for each continuous variable to assess normality. This revealed that the distributions of responses at both time points for both real world outcomes and negative affect were positively skewed while scores on the executive function task were negatively skewed. Log transformations were carried out on these variables (with scores on the executive function task being reflected first). Box plots and z-scores were examined in order to identify potential outliers. No significant outliers were identified.

**Table 3:** Means with 95% confidence intervals and standard deviations for primary and secondary measures

	Sham Meditation						Mindfulness Meditation					
	Baseline			Follow-up			Baseline			Follow-up		
	M	95% CI	SD	M	95% CI	SD	M	95% CI	SD	M	95% CI	SD
Observing	25.58	[24.06, 27.18]	5.60	27.29	[25.63, 28.93]	5.74	26.63	[25.26, 28.00]	4.83	28.42	[27.13, 29.63]	4.52
Non-reactivity	20.10	[18.87, 21.35]	4.61	21.31	[20.08, 22.60]	4.46	20.37	[18.78, 21.89]	5.08	21.91	[20.60, 23.16]	4.21
Acting with Awareness	23.98	[22.31, 25.82]	6.43	24.60	[22.85, 26.47]	6.37	24.84	[23.30, 26.40]	5.19	24.81	[23.13, 26.42]	5.29
Non-judgment	26.85	[25.06, 28.66]	6.42	28.23	[26.19, 30.14]	7.09	27.02	[24.88, 29.08]	6.24	27.84	[25.96, 29.75]	5.99
Describing	26.17	[24.36, 28.05]	6.71	26.54	[24.55, 28.42]	6.62	26.21	[24.60, 27.86]	5.41	27.77	[26.40, 29.19]	4.94
HCTA	107.81	[104.35, 111.86]	12.10	113.79	[12.01, 16.58]	14.56	107.93	[103.80, 111.78]	12.67	113.49	[108.90, 117.88]	14.90
Heuristics and Biases	7.54	[6.85, 8.31]	2.12	7.65	[2.00, 2.54]	2.30	6.95	[6.26, 7.63]	2.35	7.21	[6.56, 7.86]	2.16
Actively Open-Minded Thinking	181.52	[175.98, 187.18]	18.59	182.98	[17.94, 23.78]	21.29	174.91	[169.65, 180.70]	19.10	179.11	[173.06, 185.04]	20.37
Need for Cognition	63.79	[60.83, 66.96]	10.49	63.54	[8.17, 12.78]	10.69	58.05	[54.33, 61.41]	12.25	58.79	[54.90, 62.36]	13.36
Executive Function	51.15	[50.33, 51.94]	2.90	50.67	[2.66, 4.40]	3.58	50.49	[48.56, 51.88]	4.85	50.65	[49.75, 51.53]	2.87
Wellbeing	49.98	[48.27, 51.71]	6.58	51.15	[5.57, 7.70]	6.82	50.28	[48.21, 52.42]	6.91	53.35	[51.14, 55.65]	7.00
Positive Affect	34.08	[32.13, 35.92]	7.04	35.29	[6.01, 8.00]	7.15	33.81	[31.74, 36.00]	7.32	34.91	[32.41, 37.24]	7.33
Negative Affect	20.17	[18.15, 22.15]	7.29	19.38	[5.17, 7.19]	6.33	18.95	[16.96, 20.97]	7.34	17.65	[15.95, 19.63]	6.59
Real World Outcomes	3.71	[2.96, 4.54]	2.77	3.23	[1.76, 2.94]	2.43	3.12	[2.53, 3.79]	2.06	3.30	[2.70, 3.90]	2.08

### Manipulation checks

These analyses were carried out to investigate whether any other characteristics of the intervention besides the content may have affected its outcomes and participant adherence and whether the differences in intervention content led to differences in meditation quality. Tables 4 and 5 displays correlations between manipulation check measures and change scores for executive function and critical thinking measures. There are several relationships of note here. Changes in performance from baseline to follow-up on measures of critical thinking and executive function were not related to any manipulation check measures. There were, however, significant positive correlations observed between meditation quantity and increases in HCTA scores and observing in both groups. Meditation quantity was also positively related to task ease and enjoyment for the sham meditation group. In terms of meditation quality, while receptivity was not significantly related to any manipulation check measures in the mindfulness meditation group, there was a strong positive association between receptivity and task ease for the sham meditation group. Perseverance was positively related to satisfaction with the intervention for both groups and also to task ease within the mindfulness meditation group.

**Table 4:** Means and standard deviations for manipulation check variables and their correlations with change scores

		Sham Meditation								Mindfulness Meditation							Overall	
	N	*M*	*SD*	ΔHCTA	ΔHB	ΔSWM	ΔOBS	ΔNR	N	*M*	*SD*	ΔHCTA	ΔHB	ΔSWM	ΔOBS	ΔNR	*M*	*SD*
Meditation Quantity	48	14.85	12.52	0.53^**^	−0.03	− 0.11	0.47^**^	0.27	43	15.72	13.61	0.32^*^	−0.13	0.17	0.36^*^	0.23	15.26	12.98
Week 2 TAM Barriers	21	23.51	29.65	0.25	−0.27	− 0.29	− 0.04	− 0.07	25	28.49	32.08	− 0.07	− 0.18	− 0.03	0.42	0.07	26.22	30.75
Week 4 TAM Barriers	35	5.69	2.74	− 0.05	− 0.06	0.00	− 0.38^*^	0.02	28	4.93	1.92	0.08	0.12	−0.09	− 0.39^*^	− 0.03	5.35	2.42
Week 2 TAM Ease	21	35.97	31.44	0.17	−0.10	− 0.38	− 0.12	− 0.42	25	31.99	28.24	− 0.12	−0.23	− 0.19	0.22	0.06	33.80	29.48
Week 4 TAM Ease	35	14.00	1.41	0.29	−0.15	−0.15	0.34^*^	0.03	28	14.04	1.57	0.07	−0.12	0.37	0.21	0.00	14.02	1.48
Week 2 TAM Enjoyment	21	7.52	3.27	−0.13	−0.07	0.18	0.22	0.12	25	8.20	3.33	0.26	0.21	0.07	−0.36	−0.02	7.89	3.28
Week 4 TAM Enjoyment	35	9.57	2.34	0.08	0.00	−0.19	0.05	0.24	28	10.86	1.67	−0.08	−0.02	0.09	0.07	−0.13	10.14	2.15
Week 2 Satisfaction	21	3.76	.77	0.10	−0.15	0.04	0.12	−0.14	25	3.96	0.68	0.27	−0.11	−0.04	0.05	−0.03	3.87	0.72
Week 4 Satisfaction	35	3.50	.86	0.00	−0.14	−0.03	0.18	−0.16	28	4.10	0.43	−0.18	0.01	0.12	−0.01	0.05	3.88	0.72
Week 2 PMQ Perseverance	21	61.81	24.07	0.14	0.04	−0.10	−0.14	−0.40	25	67.04	23.17	0.16	−0.15	−0.14	0.05	0.20	64.65	23.47
Week 4 PMQ Perseverance	35	55.43	21.38	0.24	−0.13	−0.14	0.16	0.15	28	64.55	18.81	0.02	0.24	−0.13	0.11	0.14	59.48	20.63
Week 2 PMQ Receptivity	20	62.05	25.31	0.30	0.35	−0.01	−0.05	−0.18	25	70.01	21.75	0.28	−0.17	−0.02	− 0.20	0.09	66.47	23.47
Week 4 PMQ Receptivity	35	72.73	19.13	−0.05	0.15	0.08	−0.20	−0.26	28	72.39	19.00	0.16	0.13	−0.03	0.08	−0.10	72.58	18.92

Note: Δ = Change score; TAM = Technology Acceptance Model; PMQ = Practice Quality – Mindfulness; HCTA = Halpern Critical Thinking Assessment; SWM = Sternberg Working Memory Task; OBS = Observing Subscale of Five Facet Mindfulness Questionnaire; NR = Non-reactivity of Five Facet Mindfulness Questionnaire

* denotes *p* < .05

** denotes *p* <.01

**Table 5:** Correlations of manipulation check variables for sham meditation and mindfulness meditation groups

	1	2	3	4	5	6	7	8	9	10	11
1. Meditation Quantity	1	−0.16	0.06	0.21	0.00	0.03	0.28	−0.01	0.01	0.16	0.16
2. Week 2 TAM Ease	0.60^*^	1	0.56	0.19	0.31	0.23	0.38	0.66^**^	0.39	−0.13	0.52
3, Week 4 TAM Ease	0.59^**^	0.59	1	−0.35	0.20	−0.36	−0.03	0.16	0.24	0.21	0.08
4. Week 2 TAM Enjoyment	0.01	0.23	0.22	1	0.31	0.42	0.38	0.32	−0.06	0.33	0.45
5. Week 4 TAM Enjoyment	0.34^*^	0.50	0.58^*^	0.56	1	−0.04	0.12	0.00	−0.29	0.22	0.14
6. Week 2 Satisfaction	0.09	0.31	0.09	0.76^**^	0.39	1	0.28	0.57^*^	0.02	0.40	0.32
7. Week 4 Satisfaction	0.13	−0.18	0.28	0.47	0.38^*^	0.66^*^	1	0.69^*^	0.18	0.46	0.23
8. Week 2 PMQ Perseverance	0.04	0.21	−0.25	0.24	−0.09	0.36	0.25	1	0.71^*^	0.02	0.29
9. Week 4 PMQ Perseverance	0.16	−0.51	0.26	−0.24	0.27	−0.42	0.34^*^	0.90^**^	1	0.35	−0.07
10. Week 2 PMQ Receptivity	0.25	0.33	0.03	0.01	−0.12	0.44	−0.08	0.20	−0.01	1	0.73^**^
11. Week 4 PMQ Receptivity	−0.13	0.70^*^	−0.07	−0.59	− 0.21	−0.13	− 0.19	−0.24	− 0.45^**^	0.73^*^	1

Note: Bottom Left quadrant = Correlations for Sham Meditation Group. Top Right quadrant = Correlations for Mindfulness Meditation Group. TAM = Technology Acceptance Model; PMQ = Practice Quality – Mindfulness

Only 23 participants completed the manipulation check measures at both time points. It was decided that this sample size was insufficient to justify the analysis of differences across time and therefore, contrary to pre-registered analyses, only group differences at each time point are reported. Differences between the mindfulness meditation and sham meditation groups on each of the manipulation check measures at each time point were analysed using a series of independent t-tests.

There was no evidence for a difference between the groups in meditation quantity and, on average, participants completed half of the 30 sessions they were asked to complete (*t*(89) = − 0.32, *p* = 0.75). Table 6 breaks down this average to show that a third of the sample did not complete any meditation sessions, half of the sample completed at least half of the sessions and a quarter of the sample completed all 30 sessions.

**Table 6:** Percentage of sessions completed by the overall sample and each group separately

	None	At least 1	At least 10	At least 15	At least 20	Complete
Overall	31.87	68.13	58.24	50.55	37.36	24.18
Sham Meditation	35.42	64.58	60.42	54.17	37.50	18.75
Mindfulness Meditation	27.91	72.09	55.81	46.51	37.21	30.23

Meditation quality consists of two factors focusing on perseverance and receptivity. At week 2 of the intervention, there was no evidence for a significant difference between the groups in either perseverance (*t*(44) = − 0.75, *p* = 0.46, Mean difference = − 5.23 [− 19.30, 8.84]) or receptivity (*t*(43) = − 1.14, *p* = 0.26, Mean difference = − 7.96 [− 22.12, 6.19]). Similarly, at week 4, no evidence was found for a significant difference between the groups in terms of perseverance (*t*(61) = − 1.77, *p* = 0.08, Mean difference = − 9.12 [− 19.40, 1.16]) or receptivity (*t*(61) = .07, *p* = 0.94, Mean difference = 0.34 [− 9.33, 10.01]).

Subscales from the TAM were used to assess the extent to which participants found the Headspace app enjoyable and easy to use. There was no evidence found for a difference in terms of either enjoyment (*t*(44) = − 0.69, *p* = 0.49, Mean difference = − 0.68 [− 2.65, 1.29]) or perceived ease (*t*(44) = 0.45, *p* = 0.65, Mean difference = 3.98 [− 13.76, 21.72]) at week 2. At week 4, there was a significant difference between the groups in their enjoyment of Headspace (*t*(61) = − 2.54, *p* = 0.01, Mean difference = − 1.29 [− 2.30, − 0.27]) with the sham meditation group (*M* = 9.57, *SD* = 2.34) enjoying it slightly less than the mindfulness meditation group (*M* = 10.86, *SD* = 1.67). There was no evidence for a significant difference in perceived ease of use at week 4 (*t*(61) = − 0.10, *p* = 0.93, Mean difference = − 0.04 [− 0.79, 0.72]).

Evidence for a difference between the groups in overall satisfaction with the intervention was not found at week 2 (*t*(44) = − 0.93, *p* = 0.36, Mean difference = − 0.20 [− 0.63, 0.23]). However, at week 4 there was a significant difference (*t*(58.63) = − 2.08, *p* = 0.04, Mean difference = − 0.36 [− 0.70, − 0.01]) which suggested that the mindfulness meditation group (*M* = 4.07, *SD* = 0.53) were slightly more satisfied than the sham meditation group (*M* = 3.71, *SD* = 0.83).

Finally, logistic regressions were carried out to examine what factors (at each measurement point), including meditation quality and quantity, and task enjoyment and difficulty, predicted participant attrition. The model including these factors as measured during week 2 of the intervention was statistically significant and correctly classified 96% of cases (χ^2^(5) = 16.39, *p =* 0.006) but none of the factors individually predicted attrition. Later in the intervention participants who reported greater enjoyment using Headspace and those who completed more meditation sessions were more likely to attend follow-up data collection. This model, which included the same variables as measured during week 2 of the intervention classified 95% of cases correctly (χ^2^(5) = 38.28, *p* < 0.001). See Table 7 for a summary of these models.

**Table 7:** Logistic regressions predicting participant attrition

	β	SE	Wald	*df*	*p*	Odds Ratio	95% CI for Odds Ratio	
Week 2							Lower	Upper
Meditation Quantity	−2.27	255.19	0.00	1	0.99	0.10	0.00	1.69 × 10^199^
PMQ Perseverance	−0.01	0.05	0.08	1	0.78	0.99	0.90	1.08
PMQ Receptivity	0.02	0.08	0.07	1	0.79	1.02	0.88	1.19
TAM Ease	1.93	1.54	1.57	1	0.21	6.86	0.34	139.47
TAM Enjoyment	−1.64	1.59	1.07	1	0.30	0.19	0.01	4.34
Week 4								
Meditation Quantity	−0.53	0.23	5.26	1	0.02	0.59	0.38	0.93
PMQ Perseverance	0.02	0.05	0.17	1	0.68	1.02	0.92	1.13
PMQ Receptivity	0.10	0.06	2.98	1	0.08	1.11	0.99	1.24
TAM Ease	1.40	0.99	1.99	1	0.16	4.06	0.58	28.47
TAM Enjoyment	−1.44	0.72	3.95	1	0.05	0.24	0.06	0.98

### Primary analyses

Hypothesis 1 stated that mindfulness would increase more for the mindfulness meditation group than for the sham meditation group from baseline to follow-up. As can be seen in Table 8, all aspects of mindfulness increased for both groups from baseline to follow-up, group assignment was not associated with change in any of the facets of dispositional mindfulness. Therefore this hypothesis is not supported.

**Table 8:** Models testing the effect of group allocation on facets of dispositional mindfulness while controlling for baseline measures of each facet

	*b*	*SE*	*p*	95% CI for *b*	
				Lower	Upper
Observing					
Group	0.26	1.02	0.80	− 1.76	2.29
Baseline	0.59	0.10	< 0.001	0.39	0.78
Non-reactivity					
Group	0.67	0.85	0.92	−1.02	2.36
Baseline	0.39	0.09	< 0.001	0.21	0.57
Non-judgment					
Group	−0.90	1.26	0.47	−3.40	1.60
Baseline	0.65	0.10	< 0.001	0.45	0.85
Acting with Awareness					
Group	−0.98	0.92	0.29	−2.81	0.85
Baseline	0.68	0.08	< 0.001	0.52	0.81
Describing					
Group	1.24	0.83	0.14	−0.63	4.65
Baseline	0.76	0.07	< 0.001	0.62	0.90

Hypothesis 2a^1^ stated that critical thinking as measured by the HCTA would increase more for the mindfulness meditation group than for the sham meditation group from baseline to follow-up. The effect of group assignment was small and not statistically different from 0 (b = − 1.52 [− 6.26, 3.22], *p* = 0.53). Therefore this hypothesis is not supported. Hypothesis 2a^2^ stated that critical thinking as measured by items from the heuristics and biases literature would increase more for the mindfulness meditation group than for the sham meditation group from baseline to follow-up. Group assignment did not have a significant effect on performance on these items at follow-up after controlling for baseline levels of performance (b = − 0.13 [− 0.90, 0.64], *p* = 0.74). Hypothesis 2b stated that the above effects would be moderated by levels of need for cognition and actively open-minded thinking respectively. Hypothesis 2b^1^ was not supported as the interaction between group assignment and need for cognition was not associated with performance on the HCTA at follow-up (b = 0.18 [− 0.24, 0.60], *p* = 0.39). Similarly, no moderation effect was found for actively open-minded thinking (b = − 0.06 [− 0.30, 0.19], *p* = 0.65). Hypothesis 2b^2^ was not supported due to the lack of evidence for the moderation of the effects of the intervention on the heuristics and biases items by either need for cognition (b = 0.04 [− 0.03, 0.10], *p* = 0.25) or actively open-minded thinking (b = − 0.02 [− 0.06, 0.03], *p* = 0.46).

Hypothesis 3 stated that need for cognition and actively open-minded thinking would increase more for the mindfulness meditation group than for the sham meditation group from baseline to follow-up. Need for cognition at follow-up was not associated with group assignment after controlling for its measurement at baseline (b = 0.85 [− 1.91, 3.61], *p* = 0.54). Group assignment also did not affect levels of actively open-minded thinking at follow-up (b = 2.90 [− 2.34, 8.15], *p* = 0.27).

Hypothesis 4a stated that executive function would increase more for the mindfulness meditation group than for the sham meditation group from baseline to follow-up. Hypothesis 4b stated that there would be an indirect effect of group allocation on critical thinking through executive function. No differences in performance on the executive function task at follow-up were found between conditions, after controlling for baseline performance (b = 0.02 [− 0.12, 0.16], *p* = 0.77). Furthermore, when simple mediation models were run in AMOS, the bootstrapped 95% confidence intervals for the indirect effects of group allocation on performance on the HCTA (*b* = − 0.42, 95% CI [− 3.18, 2.30], *p* = 0.75) and the heuristics and biases items (*b* = − 0.03, 95% CI [− 0.27, 0.14], *p* = 0.57) through executive function included 0. Therefore neither of these hypotheses were supported.

### Secondary analyses

Hypothesis 5 stated that wellbeing would increase more across time for the mindfulness meditation group. Group assignment was not associated with wellbeing at follow-up after baseline wellbeing was controlled for (*b* = 2.01, 95% CI [− 0.63, 4.65], *p* = 0.13).

Hypothesis 6 stated that positive affect would increase more across time for the mindfulness meditation group while negative affect would decrease more for this group. Neither positive affect (*b* = − 0.61, 95% CI [− 3.45, 2.23], *p* = 0.67) nor negative affect (*b* = − 1.03, 95% CI [− 3.22, 1.16], *p* = 0.35) changed as a function of group assignment.

Hypothesis 7 stated that the number of recent negative life events would reduce to a greater extent for the mindfulness meditation group. However, this hypothesis was not supported (*b* = 0.39, 95% CI [− 0.44, 1.22], *p* = 0.36).

### Additional exploratory analyses

In order to whether the extent to which participants engaged in the intervention was a factor in the results reported above, further exploration of the data was carried out in two ways – a per protocol analysis of the hypotheses tested above and analyses with meditation quantity as a moderator. The per protocol analysis includes only those participants that attended both baseline and follow-up. Seventy-five percent of this sub-sample of participants completed at least half of the 30 meditation sessions. These analyses followed exactly the same pattern as those reported above and due to this and their exploratory nature, it was deemed unnecessary to report these findings individually. The moderation analyses were carried out in AMOS and estimates were computed using the full information maximum likelihood method. These models included group allocation as the independent variable and the various outcomes measured at the end of the intervention as the dependent variable. Individual baseline differences were controlled for by including the outcomes as measured at baseline as a covariate in each model. The interaction between group allocation and meditation quantity was included to test for moderation. This interaction effect was not significant for any of the primary or secondary outcomes indicating that there were no changes in any outcome due to increased engagement with meditation sessions.

## Discussion

This study was designed to investigate the claim that mindfulness practice improves critical thinking. This claim was tested by randomly allocating carefully screened volunteers to either a mindfulness meditation program or a closely matched active-control condition for 6 weeks. Differences in performance, across time and both groups, on an established critical thinking measure, items from the literature on heuristics and biases, key thinking dispositions and executive function were examined. It also tested whether executive function mediates the relationship between mindfulness and critical thinking in line with default interventionist theory and previous cross-sectional and experimental studies which examined this relationship. Secondary analyses examined the effects of mindfulness practice on wellbeing, affect and life outcomes.

Our results show that, for most outcomes, there were significant changes from baseline to follow-up but none which can be specifically attributed to the practice of mindfulness. Looking at dispositional mindfulness, one can see that participants from both conditions endorsed each facet to a greater extent at the end of the intervention. This could be due to insensitivity of the measure employed, a genuine small effect of the active-control condition on dispositional mindfulness or simply that low intensity guided practice of mindfulness meditation is no more effective in training the skills of mindfulness than the sham meditation condition we employed. Indeed, critics might argue that we should not expect people to learn how to practice mindfulness just by using a smartphone application and without facilitator involvement [88, 89]. However, it must be acknowledged that it is currently one of the most popular methods for doing so and it appears to be effective in some cases, at least for reducing anxiety, depression and stress [53]. There are many studies using guided meditations similar to those in our mindfulness meditation condition, delivered through smartphone applications [49, 50, 52, 90, 91], websites [92–97] and CDs [98, 99], which show effects on measures of outcomes reliably associated with increases in mindfulness such as depression, anxiety, stress, wellbeing and compassion. There are two things to note about these studies – they tend not to include a measure of dispositional mindfulness (e.g. only 4% of all mindfulness intervention studies reviewed in a recent meta-analysis include such measures at baseline and follow-up; [54]) and they usually employ a weak form of control group such as a no-treatment control or waitlist control [54]. Therefore, even when change in mindfulness is assessed in mindfulness meditation intervention studies, it is usually overestimated and this must be borne in mind when comparing the results of this study with those of previous studies. This combined with generally only moderate correlations with behavioural outcomes [54] suggests that when mindfulness interventions are effective, dispositional measures do not fully capture what has changed. This means the results of the effects of the current intervention on dispositional mindfulness are not conclusive due, in part, to the poor sensitivity of the dispositional mindfulness measure. This poor sensitivity may undermine the use of sham meditations as an active control as the wording of the items could be in line with the expectations of participants who think they are engaging in meditation, particularly given the current ubiquity of information about meditation and mindfulness. While some evidence for a distinction between the effects of the experimental and control conditions can be seen in the difference in receptivity during initial meditation sessions, it is clear that more studies evaluating the Headspace intervention materials are required in order to evaluate its efficacy for increasing mindfulness. These results align with those of a recent study using mobile devices to deliver a mindfulness meditation intervention or a sham meditation intervention which also included much more fine-grained data by including multiple momentary assessments during each day of the intervention [100]. While this study also found no changes in trait mindfulness or cognition which could be attribute to the mindfulness intervention, analysis of the momentary data using linear mixed models showed that state mindfulness increased to a greater extent over time for the mindfulness meditation group. Future research evaluating the Headspace intervention materials should take this innovative approach to data collection and analysis.

If the mindfulness meditation intervention did indeed succeed in increasing mindfulness somewhat, this did not result in a greater increase in critical thinking than the sham meditation condition did. While performance on the HCTA improved somewhat from baseline to follow-up for both groups, no such improvement was found for performance on the heuristics and biases items. Performance on the executive functioning measure was stable across both groups and time and there was no evidence of conditional effects dependent on thinking dispositions or an indirect effect on critical thinking through executive functioning. Furthermore, there were no significant group related changes in actively open-minded thinking or need for cognition and baseline levels of these thinking dispositions did not moderate the effect of the intervention. While previous mindfulness intervention studies have demonstrated positive effects on related measures such as insight problem-solving [27, 47], moral decision-making [29] and cognitive rigidity [48], none of these studies employed an active control group and so whether these effects are due solely to mindfulness practice is unclear. Each of these studies claimed the inhibition of automatic responses as the likely mechanism underlying these effects but did not test for this. Subsequent research focusing specifically on critical thinking demonstrated that its relationship with dispositional mindfulness was positive and mediated by inhibition (for both observing and non-reactivity), which supported this view. However, it was also complicated by the existence of a negative direct effect of non-reactivity on critical thinking [3]. In the current study, engaging in a 6 week long mindfulness meditation intervention did not improve critical thinking or executive functioning to a greater extent than a closely matched active control condition. This occurred despite quite good adherence to the experimental procedure by participants in both conditions. This suggests that the aforementioned studies on mindfulness and higher-order cognition may have overestimated this relationship. While further research is warranted to examine whether more intensive mindfulness meditation interventions can enhance critical thinking, it appears that one of the most common methods for learning mindfulness meditation does not do so.

This method for learning mindfulness meditation also failed to significantly affect emotional experience or wellbeing when compared to the sham meditation condition. Two previous studies employing Headspace where participants engaged in *less* meditation sessions did report positive effects on wellbeing and positive affect [49, 50]. However, these effects were relatively small and resulted from comparisons with a waitlist control group and a poorly matched active control group respectively. Therefore conclusions in these studies regarding the efficacy of Headspace appear to be premature. Though participants in the current study engaged in more meditation sessions, we did not observe significantly better outcomes for wellbeing, positive affect or negative affect for those in the mindfulness meditation group and this was likely due to how well matched the sham meditation active control condition was. Only one other study comparing a mindfulness meditation condition to a sham meditation condition has examined changes in negative affect over time. This study showed that negative affect decreased to greater extent for those in the mindfulness meditation group [58]. However, this intervention lasted only 3 days and so is not comparable to that of the current study. Finally, there was an increase in wellbeing and a decrease in negative affect for both groups which could have been due to expectation effects and/or genuine positive effects of the relaxed breathing common across both intervention conditions [101]. Again, whether a more intensive or long-term application of an online mindfulness intervention would prove effective in improving emotional experience and wellbeing is an open empirical question.

This was a methodologically rigorous study. Potential bias was reduced by pre-registering the study, publishing a study protocol [61] and blinding both the experimenters and participants to group allocation. An active-control group was employed in order to avoid the artificial inflation of effect sizes observed in the application of waitlist control groups [42]. Finally, socially desirable reporting of intervention adherence was avoided by measuring the number of intervention sessions engaged in by the participants directly through the Headspace application rather than by self-report.

There are weaknesses with this study also. While it was intended that the only difference between the experimental and the active-control conditions would be the provision of specific instructions to do with building specific mindfulness skills in the guided mindfulness meditations, this was not possible in practice as Headspace only provided one guided sham meditation recording. Therefore, another key difference between the conditions was the variability in the content as this guided sham meditation was repeated for each session completed by those in the control group. This was in contrast to the progressive nature of the guided mindfulness meditations. The fact that we failed to observe increases specific to the mindfulness meditation group in dispositional mindfulness or measures of wellbeing and emotional experience usually associated with mindfulness practice raises the possibility that this intervention was not effective and that similar previous studies did not really raise levels of mindfulness (i.e. significant effects may be an artefact due to high sample sizes and poorly matched control groups). If this is the case, we cannot reasonably conclude anything about the relationship between mindfulness and critical thinking except that this approach to learning mindfulness does not enhance either dispositional mindfulness or critical thinking. It is also possible that the active nature of the control condition, and its similarity to the intervention condition, had a beneficial effect on mindfulness and other primary outcomes. Future studies could also include an active control condition which is similar in delivery but obviously not meditation, audiobooks for example. This would allow the disentanglement of effects due to specific mindfulness instruction, and effects due to sham mindfulness instruction, from the effects of a listening activity which does not carry the expectation of relaxation. In terms of measurement, a major limitation of this study is the operationalisation of executive control of working memory. While the Sternberg memory task is a recognised test of working memory resources, it does not require a great deal of control per se. Therefore, a cognitive task specifically focused on inhibition would have been a stronger test of the theoretical framework applied in this study since it is the inhibition of type-1 processes which is the mechanism by which type-2 processes intervene [25, 34]. In addition, the inclusion of a more objective measure of mindfulness such as the recently developed meditation breath attention scores task would have strengthened our measurement approach also [102]. Finally, the results of this study are not generalizable to the general population as the sample solely consisted of university students. Still, it has been claimed that introducing mindfulness programmes in universities may improve critical thinking – a particularly important outcome of university education – so the focus on the university context was deemed to be important [2]. It should be noted however that the incentives to participate, including study credit, a free subscription to Headspace and refreshments, may have affected the study results. It is likely that many participants were not motivated to a large extent by interest in practicing mindfulness. This may have affected engagement with the intervention materials and contributed to the null effect found. It also underlines the importance of careful investigation of the feasibility and acceptability of mindfulness interventions prior to examinations of their effectiveness.

Several recommendations for future research arise out of the current study. A priority for the field of mindfulness research should be the development of better measures. Few studies, the current one included, show significant increases in dispositional mindfulness following intervention which suggests that the measures available are not sensitive enough [54]. Ideally, objective measures which can capture the full mindfulness would be developed and used. Current objective measures focus primarily on the present-moment attention aspect of mindfulness [102, 103]. It is possible that with a longer or more intensive mindfulness intervention, and/or the involvement of a mindfulness instructor, that changes in dispositional mindfulness and critical thinking may have been observed. More research of this sort is needed before the findings of the current study can be confirmed. As the relationships between dispositional mindfulness, state mindfulness and cognition is complex [104], future studies should plan to recruit adequate sample sizes to allow mediation and moderation analyses and SEM to disentangle these relationships. It also would have been useful to have manipulated state mindfulness at follow-up to examine whether being in a state of mindfulness at the time of measurement is necessary to observe the effects of a mindfulness intervention and future studies should consider this approach.

## Conclusions

To summarise, despite claims regarding the benefits of mindfulness practice for everyday thinking skills, few studies have shown evidence for this relationship, and those which have are not without significant limitations. This study was designed to rigorously test the effects of regular mindfulness practice on critical thinking performance in a sample of university students who had never practiced mindfulness meditation before. No evidence was found to suggest that engaging in guided mindfulness practice for 6 weeks, using the online intervention method applied in this study, improves critical thinking performance. While further research is warranted, claims regarding the benefits of mindfulness practice for critical thinking should be tempered until evidence of these supposed benefits are presented.

## Additional file

Additional file 1: A. Headspace Meditation Intervention Components. A detailed description of the materials used in each intervention condition. B. Analyses Originally Specified in Study Protocol. In the interest of transparency, all analyses specified in the pre-registered protocol are reported. (DOCX 25 kb)

## Abbreviations

AMOS: Analysis of a moment structures
ANOVA: Analysis of variance
CDs: Compact Discs
CONSORT: Consolidated Standards of Reporting Trials
FFMQ: Five Facet Mindfulness Questionnaire
GLB: Greatest lower bound
HCTA: Halpern critical thinking assessment
IELTS: International English Language Testing System
ISRCTN: International Standard Registered Clinical/Social Study Number
OASAS: Office of alcoholism and substance abuse services
PANAS: Positive and negative affect schedule
PMQ: Practice Quality- Mindfulness questionnaire
RCT: Randomised controlled trial
SEM: Structural equation modelling
SPSS: Statistical Package for the Social Sciences
TAM: Technology acceptance model
TOEFL: Test of English as a Foreign Language

## Footnotes

1 Hypotheses 1–7 were also analysed through a series of 2 × 2 (time – pre, post x group – mindfulness meditation, sham meditation) mixed ANOVAs for each outcome measure in accordance with the pre-registered analysis strategy. The results for these analyses are presented as Additional file 1. The full information maximum likelihood-estimated regression analyses presented in this manuscript were conducted on the advice of a reviewer as this approach to handling missing data produces more accurate estimates than imputing the baseline measurement and allows for greater statistical power than the mixed ANOVA.
