# Supplementary material for: A randomised active-controlled trial to examine the effects of an online mindfulness intervention on executive control, critical thinking and key thinking dispositions in a university student sample
Source: BMC Psychol. 2018 Apr 5;6:13. doi: 10.1186/s40359-018-0226-3 (PMC5887193; doi:10.1186/s40359-018-0226-3)
Supplement: Supplementary file 1 — A. Headspace Meditation Intervention Components. A detailed description of the materials used in each intervention condition. B. Analyses Originally Specified in Study Protocol. In the interest of transparency, all analyses specified in the pre-registered protocol are reported. (DOCX 25 kb) [file 40359_2018_226_MOESM1_ESM.docx]

**Supplementary Material**

**A. Headspace Meditation Intervention Components**

Headspace Session 1

The first session begins with advice on the practicalities of meditating including how to build a routine regarding where to meditate, what time to meditate and the attitude to bring to meditation. The guided meditation follows the following steps:

1. The guided meditation begins with instructions on getting into a comfortable seated position and taking deep breaths with the eyes open. (Approx. 30 seconds)
2. The participant is then invited to notice the weight of their body against the chair and other physical sensations. (Approx. 30 seconds)
3. The participant is guided through a scan of their body from head to toe and back up to the chest where the focus of the participant is guided towards the breath. (Approx. 1 minute)
4. The participant is then instructed to anchor their attention to the breath by counting each breath, starting at 1 and ending at 10 before repeating the cycle. During this time, the participant is encouraged to monitor for mind-wandering and to gently return attention to the breath when mind-wandering is detected. (Approx. 7 minutes)
5. The participant is then allowed to let their mind wander before bringing their attention back to their body and their surroundings to end the meditation. (Approx. 1 minute)

Headspace Session 2

This session and each of the subsequent sessions follow the same sequence as the guided meditation in the first session but with subtle differences which will be noted below. For example, this session gives more time to the breath counting exercise.

Headspace Session 3

This session begins with an animation explaining how to monitor thoughts and feelings in the present moment by using a metaphor of watching passing cars from the side of a road.

Headspace Session 4

This session begins with an introduction which encourages the participant to bear in mind the metaphor presented in the previous session and then continues with the guided meditation.

Headspace Session 5

An animation is presented at the beginning of this session which discusses the role of effort in mindfulness meditation, where it is suggested that exerting too much effort is detrimental to the practice. This point is made using the metaphor of taming a wild horse.

Headspace Session 6

In this session, the guided meditation is presented in a similar way to previous sessions but with extra emphasis put on the body scan exercise. Participants are encouraged to try their best to not resist unpleasant feelings during this exercise.

Headspace Session 7

This session starts with an animation which discusses detachment from unpleasant thoughts and feelings. This is elaborated upon by comparing a calm state of mind to a clear sky and a stressed state to a stormy sky. Detachment is compared to the ability to fly high enough away from the clouds so that the sky is again clear. The guided mediation then proceeds per the same sequence as previous sessions, though more periods of silence (and less instructions) are included.

Headspace Session 8

This session begins with the instruction to pay attention specifically to one’s mood state during the guided meditation.

Headspace Session 9

At the start of this session, an animation is presented which compares the mind to a still pool of water which can be disturbed, but always has the potential to return to being still. This metaphor is explored further in order to explain the importance of allowing thoughts and feelings to come and go, rather than resisting them.

Headspace Session 10

At the start of this session, the participant is encouraged to reflect on what differences they have noticed since they began the series of meditation sessions.

Headspace Session 11

This session begins with a video in which the instructor, Andy Puddicombe, reminds participants of the tips presented in the first session regarding how to approach the guided meditations. It then proceeds with the same steps as previous guided meditations but in this and the following sessions, there is gradually longer periods of silence and less instruction during the breath counting exercise.

Headspace Session 12

This session begins with the participant to reflect on how their motivation to meditate relates to how their meditation practice affects others around them.

Headspace Session 13

At the beginning of this session, participants are reassured about the difficulty they may be experiencing in motivating themselves to meditate and encouraged to be non-judgmental towards themselves.

Headspace Session 14

A similar reminder to be non-judgmental regarding one’s motivation to meditate is presented at the beginning of this session.

Headspace Session 15

This session begins with a video which includes tips on integrating mindful attention into daily activities. It then discusses the barrier of restlessness during meditation and encourages participants to not react to this feeling.

Headspace Session 16

This session begins with a discussion of the barrier to practice of sleepiness during meditation with the instruction to notice any resistance to sleepiness during the guided meditation.

Headspace Session 17

This session begins with a discussion of the barrier to practice of boredom during meditation with the instruction to approach the guided meditation with a curious attitude.

Headspace Session 18

This session begins with a discussion of the barrier to practice of pain or discomfort during meditation with the instruction to give up resistance to these feelings.

Headspace Session 19

This session begins with a discussion how daydreaming, even if pleasant, can be a barrier to meditation. Participants are encouraged to monitor for any instances of daydreaming and to gently direct their attention back to the present moment when daydreaming is noticed.

Headspace Session 20

This session starts with a video in which Andy Puddicombe congratulates the participant for completing 20 sessions. He then advises participants to think about their relationships with others when considering their motivation to practice mindfulness before the guided meditation begins.

Headspace Session 21

At the beginning of this session a video is presented featuring Andy Puddicombe discussing the barriers to meditation considered in previous sessions. He also discusses the integration of mindful attention into daily activities and explains that common to both issues is the need to engage with difficult feelings as they arise rather than resist them.

Headspace Session 22

At the end of this session and subsequent sessions, participants are encouraged to monitor their breath without counting it. Participants are instructed to monitor for any instances of mind-wandering during this time.

Headspace Session 23

This session starts with tips on how to remember to pay mindful attention during the day by placing post-it notes or other physical cues in places where they will be noticed.

Headspace Session 24

At the start of this session, participants are encouraged to plan specific times during the day at which they will engage in a short body scan exercise, recalling the instructions from the guided meditations.

Headspace Session 25

This session begins with a video in which Andy Puddicombe gives advice on how to apply the body scan exercise at different times during the day by using physical cues other than the breath such as steps when walking or the feeling of heat upon one’s skin.

Headspace Session 26

The beginning of this session focuses on encouraging the participant to reflect on how they have been applying mindful attention in their everyday life.

Headspace Session 27

At the beginning of this session, participants are advised that a useful cue for initiating the engagement of present-moment attention is the transition between sitting and standing.

Headspace Session 28

This session begins with participants being asked to reflect on whether using the transition between sitting and standing as a cue to pay attention to the present moment was useful.

Headspace Session 29

The start of this session reiterates the importance of applying mindfulness during daily activities and suggests that this can help with engagement during the guided meditations.

Headspace Session 30

At the start of this session, a video is presented which features Andy Puddicombe congratulating the participant on reaching the final session.

**B. Analyses Originally Specified in Study Protocol**

In the interest of transparency, all analyses specified in the pre-registered protocol are reported here.

Manipulation Checks

Differences across group and time were analysed using a series of mixed ANOVAs. Different patterns of effects were found for both aspects of meditation quality, perseverance and receptivity. No significant effects were found for time (*F*(1, 20) = 0.04, *p* =0.84, *η*_p_^2^ = 0.002 [0.00, 0.07]), group (*F*(1, 20) = 1.59, *p* = 0.22, *η*_p_^2^ = 0.07 [0.00, 0.28]) or their interaction for perseverance (*F*(1, 20) = 0.03, *p* = 0.86, *η*_p_^2^ = 0.002 [0.00, 0.06]). The main effect of time (*F*(1, 20) = 0.01, *p* = 0.93, *η*_p_^2^ = 0.001 [0.00, 0.02]) was not significant for receptivity but there was an overall difference between the groups (*F*(1, 20) = 5.47, *p* = 0.03, *η*_p_^2^ = 0.22 [0.01, 0.43]) and an interaction effect was found (*F*(1, 20) = 9.04, *p* = 0.01, *η*_p_^2^ = 0.31). While there was a significant difference between the groups towards the beginning of the intervention (*t*(10.23) = -3.91, *p* = 0.01) such that receptivity was higher in the mindfulness meditation group (*M* = 83.50) than the sham meditation group (*M* = 58.17), this difference was not present later in the intervention (*t*(20) = -0.72, *p* = 0.48) as receptivity increased for those in the sham meditation group (*M* = 68.13; *t*(9) = -2.04, *p* = 0.07) and decreased for those in the mindfulness meditation group (*M* = 74.11; *t*(11) = 2.21, *p* = 0.05), though neither change was significant.

There was no evidence of any difference in the extent to which both groups enjoyed using Headspace, as demonstrated by the non-significant effects of time (*F*(1, 21) = 1.03, *p* = 0.32, *η*_p_^2^ = 0.05 [0.00, 0.24]), group (*F*(1, 21) = 2.16, *p* = 0.16, *η*_p_^2^ = 0.09 [0.00, 0.30]) and their interaction (*F*(1, 21) = 0.01, *p* = 0.95, *η*_p_^2^ = 0.00 [0.00, 0.02]) on enjoyment measures. Though the main effects of time (*F*(1, 21) = 0.11, *p* = 0.74, *η*_p_^2^ = 0.01 [0.00, 0.13]) and group (*F*(1, 21) = 1.87, *p* = 0.19, *η*_p_^2^ = 0.08 [0.00, 0.29]) were not significant, an interaction effect was found for task difficulty (*F*(1, 21) = 6.17, *p* = 0.02, *η*_p_^2^ = 0.23 [0.02, 0.44]) which showed that the sham meditation group (*M* = 14.73) initially found Headspace slightly easier to use than the mindfulness meditation group did (*M* = 13.25; *t*(18) = 2.83, *p* = 0.01). There was a difference in overall acceptance of the intervention, with those in the mindfulness meditation group (*M* = 4.25) reporting greater satisfaction on average than those in the sham meditation group (*M* = 3.82; *F*(1, 21) = 5.60, *p* = 0.03, *η*_p_^2^ = 0.21 [0.01, 0.42]). Time (*F*(1, 21) = 4.35, *p* = 0.05, *η*_p_^2^ = 0.17 [0.00, 0.38]) and its interaction with group (*F*(1, 21) = 0.25, *p* = 0.62, *η*_p_^2^ = 0.01 [0.00, 0.16]) had no effect on satisfaction.

*Primary Analyses*

Hypothesis 1 stated that mindfulness would increase more for the mindfulness meditation group than for the sham meditation group from baseline to follow-up. Mixed 2 X 2 ANOVAs were carried out to test this hypothesis for each facet of mindfulness. As explained above, the main focuses of this analysis were the observing and non-reactivity facets and this hypothesis would only have been considered as supported if significant interaction effects were demonstrated for at least these two facets. As can be seen in table 8, all aspects of mindfulness increased for both groups from baseline to follow-up, except for acting with awareness. However, no significant interaction effects were found. Therefore this hypothesis is not supported.

Table 8. Mixed ANOVAs testing the effects of group allocation, time and their interaction on dispositional mindfulness.

|  | *F* (1, 89) | *p* | *η*_p_^2^ | 90% CI for *η*_p_^2^ | |
| --- | --- | --- | --- | --- | --- |
|  |  |  |  | Lower | Upper |
| *Observing* |  |  |  |  |  |
| Group | 1.19 | 0.28 | 0.01 | 0.00 | 0.08 |
| Time | 14.24 | 0.0003 | 0.14 | 0.04 | 0.25 |
| Interaction | 0.01 | 0.93 | 0.0001 | 0.00 | 0.005 |
| *Non-reactivity* |  |  |  |  |  |
| Group | 0.25 | 0.62 | 0.003 | 0.00 | 0.05 |
| Time | 10.03 | 0.002 | 0.10 | 0.02 | 0.21 |
| Interaction | 0.14 | 0.71 | 0.002 | 0.00 | 0.04 |
| *Non-judgment* |  |  |  |  |  |
| Group | 0.01 | 0.93 | 0.0001 | 0.00 | 0.005 |
| Time | 4.08 | 0.05 | 0.04 | 0.00 | 0.13 |
| Interaction | 0.27 | 0.61 | 0.003 | 0.00 | 0.05 |
| *Acting with Awareness* |  |  |  |  |  |
| Group | 0.21 | 0.65 | 0.002 | 0.00 | 0.04 |
| Time | 0.57 | 0.45 | 0.01 | 0.00 | 0.06 |
| Interaction | 0.66 | 0.42 | 0.01 | 0.00 | 0.06 |
| *Describing* |  |  |  |  |  |
| Group | 0.28 | 0.60 | 0.003 | 0.00 | 0.05 |
| Time | 7.20 | 0.01 | 0.08 | 0.01 | 0.17 |
| Interaction | 2.70 | 0.10 | 0.03 | 0.00 | 0.11 |

Hypothesis 2a^1^ stated that critical thinking as measured by the HCTA would increase more for the mindfulness meditation group than for the sham meditation group from baseline to follow-up. Mixed 2 X 2 ANOVAs were carried out to test this hypothesis for both scores on the HCTA and the heuristics and biases items. While HCTA scores increased from baseline (*M* = 107.87) to follow-up for both groups (*M* = 113.65; *F*(1, 89) = 33.65, *p* < 0.001, *η*_p_^2^ = 0.27 [0.15, 0.39]), there were no group differences (*F*(1, 89) = 0.001, *p* = 0.97, *η*_p_^2^ < 0.001 [0.00, 0.001]) and no significant interaction effect (*F*(1, 89) = 0.05, *p* = 0.83, *η*_p_^2^ = 0.001 [0.00, 0.02]). Therefore this hypothesis is not supported. Hypothesis 2a^2^ stated that critical thinking as measured by items from the heuristics and biases literature would increase more for the mindfulness meditation group than for the sham meditation group from baseline to follow-up. Neither time (*F*(1, 89) = 1.15, *p* = 0.29, *η*_p_^2^ = 0.01 [0.00, 0.08]) nor group allocation (*F*(1, 89) = 2.16, *p* = 0.16, *η*_p_^2^ = 0.09 [0.00, 0.10]) significantly affected scores on these items and their interaction did not significantly affect scores either (*F*(1, 89) = 1.37, *p* = 0.25, *η*_p_^2^ = 0.02 [0.00, 0.08]). Hypothesis 2b stated that the above effects would be moderated by levels of need for cognition and actively open-minded thinking respectively. The time by group interaction effects for scores on both the HCTA and the heuristics and biases items did not depend on either need for cognition (*F*(2, 85) = 1.98, *p* = 0.14, *η*_p_^2^ = 0.05 [0.00, 0.12]) or actively open-minded thinking (*F*(2, 85) = 1.63, *p* = 0.20, *η*_p_^2^ = 0.04 [0.00, 0.11]).

Hypothesis 3 stated that actively open-minded thinking and need for cognition would increase more for the mindfulness meditation group than for the sham meditation group from baseline to follow-up. There were no overall differences between the conditions in actively open-minded thinking (*F*(1, 89) = 1.68, *p* = 0.20, *η*_p_^2^ = 0.02 [0.00, 0.09]) but need for cognition was slightly higher on average in the sham meditation group (*M* = 63.67) than in the mindfulness meditation group (*M* = 58.42; *F*(1, 89) = 4.81, *p* = 0.16, *η*_p_^2^ = 0.09 [0.00, 0.14]). While actively open-minded thinking increased for both groups across from baseline (*M* = 178.40) to follow-up (*M* = 181.15; *F*(1, 89) = 7.35, *p* = 0.008, *η*_p_^2^ = 0.08 [0.01, 0.17]), need for cognition remained the same (*F*(1, 89) = .20, *p* =.65, *η*_p_^2^ = 0.002 [0.00, 0.04]). There were no interaction effects found for either actively open-minded thinking (*F*(1, 89) = 1.73, *p* = 0.19, *η*_p_^2^ = 0.02 0[.00, 0.09]) or need for cognition (*F*(1, 89) = 0.83, *p* = 0.37, *η*_p_^2^ = 0.01 [0.00, 0.07]).

Hypothesis 4a stated that executive function would increase more for the mindfulness meditation group than for the sham meditation group from baseline to follow-up. Hypothesis 4b stated that there would be an indirect effect of group allocation on critical thinking through executive function. No differences in performance on the executive function task were found between conditions (*F*(1, 89) = 0.10, *p* = 0.75, *η*_p_^2^ = 0.001 [0.00, 0.04]) or across time (*F*(1, 89) = 0.49, *p* = 0.49, *η*_p_^2^ = 0.01 [0.00, 0.06]). The time by group interaction for performance on the executive function task was not significant (*F*(1, 89) = 0.003, *p* = 0.96, *η*_p_^2^ < 0.001 [0.00, 0.001]). Furthermore, when simple mediation models were run in AMOS, the bootstrapped 95% confidence intervals for the indirect effects of group allocation on performance on the HCTA (*b* = -0.41, 95% CI [-3.17, 2.32]) and the heuristics and biases items (*b* = -0.03, 95% CI [-0.29, 0.14]) through executive function included 0. Therefore neither of these hypotheses were supported.

*Secondary Analyses*

Hypothesis 5 stated that wellbeing would increase more across time for the mindfulness meditation group. A mixed ANOVA found showed no evidence to support this hypothesis as the interaction between group allocation and time did not have a significant effect (*F*(1, 89) = 2.65, *p* = 0.11, *η*_p_^2^ = 0.03 [0.00, 0.11]). There were no overall differences in wellbeing between the groups (*F*(1, 89) = 0.92, *p* = 0.34, *η*_p_^2^ = 0.01 [0.00, 0.07]) but wellbeing did increase for both groups to the same extent from baseline to follow-up (*F*(1, 89) = 13.14, *p* = 0.001, *η*_p_^2^ = 0.13 [0.04, 0.24]).

Hypothesis 6 stated that positive affect would increase more across time for the mindfulness meditation group while negative affect would decrease more for this group. Neither of the two separate mixed ANOVAs demonstrated interaction effects for either positive (*F*(1, 89) = 0.01, *p* =.91, *η*_p_^2^ = 0.0001 [0.00, 0.01]) or negative affect (*F*(1, 89) = 0.25, *p* = 0.62, *η*_p_^2^ = 0.003 [0.00, 0.05]) to support this hypothesis. Negative affect decreased slightly over time (*F*(1, 89) = 4.12, *p* = 0.05, *η*_p_^2^ = 0.04 [0.00, 0.13]) but there was no overall difference between groups (*F*(1, 89) = 1.18, *p* = 0.28, *η*_p_^2^ = 0.01 [0.00, 0.08]). There were no differences in positive affect across time (*F*(1, 89) = 3.41, *p* = 0.07, *η*_p_^2^ = 0.04 [0.00, 0.12]) or condition (*F*(1, 89) = 0.06, *p* = 0.81, *η*_p_^2^ = 0.001 [0.00, 0.03]).

Hypothesis 7 stated that the number of recent negative life events would reduce to a greater extent for the mindfulness meditation group. However, a mixed ANOVA showed that there were no significant differences in responses to the Real World Outcomes checklist due to group allocation (*F*(1, 89) = 0.10, *p* = 0.75, *η*_p_^2^ = 0.001 [0.00, 0.04]), time (*F*(1, 89) = 0.18, *p* = 0.67, *η*_p_^2^ = 0.002 [0.00, 0.04]) or their interaction (*F*(1, 89) = 1.76, *p* = 0.19, *η*_p_^2^ = 0.02 [0.00, 0.09]).
